# Nanostructured Chromium PVD Thin Films Fabricated Through Copper–Chromium Selective Dissolution

**DOI:** 10.3390/ma18040894

**Published:** 2025-02-18

**Authors:** Stefano Mauro Martinuzzi, Stefano Caporali, Rosa Taurino, Lapo Gabellini, Enrico Berretti, Eric Schmeer, Nicola Calisi

**Affiliations:** 1 Department of Industrial Engineering, University of Florence (DIEF), Via di Santa Marta n. 3, 50139 Firenze, FI, Italy; stefanomauro.martinuzzi@unifi.it (S.M.M.); stefano.caporali@unifi.it (S.C.); rosa.taurino@unifi.it (R.T.); eric.schmeer@edu.unifi.it (E.S.); 2 National Interuniversity Consortium of Materials Science and Technology (INSTM), Via Giuseppe Giusti n. 9, 50121 Firenze, FI, Italy; lapo.gabellini@unifi.it; 3 Department of Chemistry “Ugo Schiff”, University of Florence (DICUS), Via della Lastruccia n. 13, 50019 Sesto Fiorentino, FI, Italy; 4 Institute of Chemistry of OrganoMetallic Compounds (ICCOM), National Research Council (CNR), Via Madonna del Piano n. 10, 50019 Sesto Fiorentino, FI, Italy; eberretti@iccom.cnr.it

**Keywords:** PVD, thin film, chromium nanostructure, dealloying

## Abstract

This study investigates the fabrication of nanostructured chromium thin films via selective dissolution of PVD-deposited Cu–Cr thin films. The effects of the deposition parameters on the structural, chemical, and morphological properties of the films are systematically analyzed. Starting from a thin film composed of 50 wt.% chromium and 50 wt.% copper, deposited onto a substrate pre-heated to 300 °C, we demonstrate that the following dealloying process carried out in a diluted nitric acid solution yields nanostructured chromium films with high porosity, large surface area, enhanced wettability and neglectable copper content. These findings underline the critical influence of the deposition temperature and alloy composition on achieving optimal film properties.

## 1. Introduction

Thanks to their reduced thickness, from a few nanometers to several micrometers, thin films (TFs) present unique properties compared to bulk materials. Nowadays, TFs of diverse materials, ranging from metals and ceramics to polymers and semiconductors, are extensively studied and used in many fields [1,2,3]. Nanostructured TFs are promising materials in many technological fields, from electronics and optics to energy storage and biomedical applications [3,4,5,6,7]. They also have great potential in sensing technologies: the large surface area and the unique optoelectronic properties enable significant improvements in determining low concentrations of analytes by surface-enhanced Raman scattering (SERS) [8,9].

Nanoporous metallic TFs have also shown peculiar mechanical properties that can be exploited for developing a novel class of actuators. Specifically, gold and platinum films have been demonstrated to respond to electrical or thermal stimuli [10,11]. Moreover, nanoporous gold TFs have shown outstanding catalytic performance [12,13,14], while the high specific surface area increases the charge storage capacity, suggesting profitable applications in energy storage by means of advanced supercapacitors [14,15].

Vacuum deposition techniques such as physical and chemical vapor deposition (PVD and CVD, respectively), which provide high control in terms of thickness, morphology, and composition, further broaden the applications of TFs [16,17]. PVD is an umbrella term for different deposition techniques in which a solid material is vaporized in a vacuum environment and deposited onto substrates [18,19]. Several techniques exist to induce a phase transition from the solid to gaseous state, including thermal evaporation, laser ablation, and magnetron sputtering, to name just a few.

The application of PVD to metal TF deposition has recently gained increasing attention due to its advantages over classical electroplating technology. For example, PVD offers a cleaner and safer way to produce hard chromium coatings compared to galvanic processes based on the highly toxic hexavalent chromium, which is strictly regulated and is expected to be banned soon [20,21]. Moreover, a PVD system equipped with a magnetron sputtering source allows the deposition of virtually any kind of materials: alloys [22,23,24], composites [25,26], oxides, and ceramics [27,28], with a highly controlled structure and composition [6,17]. In such techniques, the plasma is generated near the target (the solid material to be deposited) and is confined by a magnetron system. The plasma is accelerated toward the target due to the electric field, and the elastic momentum of the impact is transferred to the target, causing the ejection of atoms. These target atoms travel through the chamber and reach the substrate, where they condense, forming the film [29].

On the other hand, dealloying is a powerful tool for producing nanostructured TFs from metallic materials. This process involves leaching one or more components from a film of metallic alloy, giving rise to a porous structure [30,31,32,33]. By combining these two processes, we exploit the rapid and flexible way to produce TFs via PVD with the dealloying process to develop an easily scalable method for producing nanostructured thin films.

Herein, we use this approach to copper–chromium layers obtained via magnetron sputtering followed by selective copper leaching, obtaining nanostructured chromium TFs, potentially suitable for applications requiring materials with a high specific surface area, such as catalysis and energy storage [9,10]. To achieve the formation of nanostructured chromium TFs, we chose to start with copper–chromium co-deposited films due to their complete immiscibility in the solid state [34]. This characteristic promotes the formation of separate domains of the two metals, facilitating the selective dissolution of copper. On the other hand, the only study reporting the production of nanostructured chromium TFs is the work of Yusuke Yoshii et al. [35], where the fabrication of nanostructured chromium TFs for sensing applications is reported, but without a comprehensive characterization of the resulting films. Nevertheless, in this paper, we investigate different deposition parameters, and the alloy compositions are investigated to identify the most performing nanostructured chromium films. To achieve this goal EDX, XPS, and SEM are employed to characterize the samples, while surface tension measurements are performed to assess its wettability.

## 2. Materials and Methods

The realization of the copper–chromium deposit was realized using a magnetron sputtering HEX system produced by Korvus Ltd. (High Wycombe, UK). The system was equipped with two sources: the first one working in direct current (DC) mode and the second one working in radio frequency (RF) alternate current mode. The copper target was mounted on the DC working source and the chromium one in the RF working source. The targets were purchased from nanoVision, both with a purity of 99.99% and in the form of a plate of 5 cm diameter. The targets were cleaned singularly, performing a pre-deposition sputtering, taking care in the monitoring of the sputtering rate using a quartz crystal microbalance (QCM) until a constant sputtering rate, ensuring the cleanliness of the cathode. Then, the sources were started together to obtain the co-deposition of the metals, leading to the formation of the TFs. The chamber of the system was pumped from a turbomolecular pump connected to a scroll pump to reach the pressure of 2 × 10^−3^ Pa prior of the deposition and then a 30 sccm argon flow was flowed in the chamber to reach a dynamic pressure of 6 × 10^−1^ Pa. The QCM was first used to determine the deposition rate of each source to obtain as a result the desired composition of the TFs by starting the two sources separately before starting the deposition. Finally, the sample holder presented a thermoresistance that permitted heating the substrate up to 400 °C. In the HEX system, the sources were placed at the bottom, close to the pumping system, and the sample holder was located on the top of the instrument, at about 15 cm. No bias potential was applied to the substrates, and they were kept in rotation at 10 rpm to ensure a uniform deposition. The sputtering parameter is reported in Table 1.

Six samples of Cu–Cr TFs were obtained by varying the relative amounts of copper and chromium in the alloy and the deposition parameters. The selection of the composition was based on the idea of investigating two types of thin films: the first with a low copper concentration, allowing the formation of more robust nanostructured thin films, and the second with a higher copper concentration. Two different theoretical compositions of the alloy were tested: 50 wt.% of copper and 50 wt.% of chromium and 30 wt.% of copper and 70 wt.% of chromium. In addition, three temperatures of the substrate were tested: room temperature, 150 °C and 300 °C, following the possibility of our instrument (Table 1).

The depositions were obtained on two kinds of substrates in the form of squares with a side length of approximately 2 cm: Si (100) and AISI 316L stainless steel. Silicon was used as a reference substrate and stainless steel as a possible substrate for future applications. Before the deposition, the silicon substrates were cleaned, sonicating with water and acetone. The stainless steel substrates were ground down using up to a P2500 emery paper, then they were washed with water, and finally, they were sonicated, firstly with water and then with acetone. During each deposition, two stainless steel substrates and two silicon substrates were placed on the sample holder: the first ones of both the substrates were used for the dealloying procedure, while the second ones were the reference samples.

**Figure 1:**
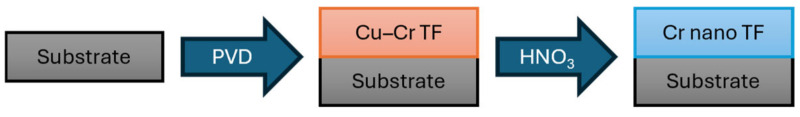
Scheme of the production process for the chromium nanostructured TFs.

The selective dissolution of copper was obtained using a diluted 1:4 solution of nitric acid (HNO_3_, purchased from Sigma-Aldrich in the form of 65% aqueous solution), following previous studies [35,36]. The samples were dipped in the acid solution for 1 h and for 24 h to verify the evolution of the relative concentration of copper and chromium. In these conditions, copper is active and should be dissolved, while chromium should be still in its passivated form. The production process of the chromium nanostructured TFs is summarized in Figure 1.

The chemical composition was obtained by means of energy dispersive X-ray analysis (EDX) using a Shimadzu Scientific Instrument (Kyoto, Japan, model EDX-7000) on the TFs deposited on the silicon substrates to prevent possible interferences with the quantification caused by the substrates. The measurements were made in air atmosphere and using a collimator of 3 mm.

X-ray photoelectron spectroscopy (XPS) was employed to determine the surface composition of the samples. The analysis was performed on the TFs deposited on the silicon substrate. The samples were fixed to the sample holder using a double-sided graphitic tape and then transferred into the XPS ultra-high vacuum (UHV) chamber. In the UHV chamber, a non-monochromatic X-ray source was positioned (VSW Scientific Instrument Limited model TA10, Manchester, UK, Mg Kα radiation, 1253.6 eV), set to work at 120 W (12 kV and 10 mA), and a hemispherical analyzer (VSW Scientific Instrument Limited model HA100) equipped with a 16-channel detector and a dedicated differential pumping system that permitted it to work during the acquisition with pressure in the main chamber up to the 10^−7^ mbar range. The pass energy was set to 44 eV for the survey scans and 22 eV for the high-definition scans. The obtained spectra were analyzed using CasaXPS (Version 2.3.25PR1.0) dedicated software. The inelastic background was subtracted using Shirley’s method [37] and mixed Gaussian and Lorentzian contributions were used for each component. The calibration of the spectra was obtained by shifting to 284.8 eV the lowest component relative to the 1s transition of carbon for adventitious carbon [38].

The surface morphologies of the samples were investigated with scanning electron microscopy (SEM). The instrument was a SU3800 scanning electron microscope (Hitachi High-Tech, Tokyo, Japan). The analysis was performed on the samples obtained on silicon substrates. Before using the sample deposited on silicon, we ensured that the substrate did not affect the superficial morphology.

The water contact angle of the films was measured by using an OCA 20 apparatus from DataPhysics Instruments GmbH, Filderstadt, Germany. Prior to the measurements, the samples were washed with acetone to prevent surface contamination. After drying, the static contact angles were measured using distilled water droplets of 5 μL volume. The final contact angle was calculated by averaging 5 measurements performed on different areas of the samples. The analysis was conducted on the samples obtained on stainless steel substrates.

The crystal structure of the TFs was investigated with X-ray diffraction (XRD) using a powder X-ray Diffractometer Anton Paar (Graz, Austria) model XRDynamic 500 in Bragg–Brentano mode. The analysis was performed on the samples obtained on silicon substrates.

Transmission electron microscopy (TEM) characterization was carried out using a Thermo Fisher (Waltham, MA, USA) TALOS F200X G2 transmission microscope, equipped with a built-in 4 elements EDS detector. The acquired images were collected using a beam energy of 200 keV. The analysis was performed on the samples obtained on silicon substrates.

## 3. Results and Discussion

### 3.1. EDX Analysis

To verify the correct deposition procedure, the compositions of the TFs were verified with EDX analysis. The results are reported in Table 2, together with the expected ones. The data related to the sample with the theoretical composition of 30 wt.% of copper and 70 wt.% of chromium are not available because the thin film did not adhere to the surface of the substrate.

As it is possible to note from Table 2, the composition of the sample Cu50-Cr50 RT is far from the expected one, suggesting that something was wrong with this sample. Anyhow, as is reported further ahead, the TFs deposited at room temperature show poor adhesion toward the substrate, causing detachment during the dealloying procedure. Considering that, we decided to not prepare the sample with the wrong composition. In general, all the samples showed a low amount of chromium compared to the expected ones, and a consequent high percentage of copper. These differences can be attributed to the different behavior of the sputtered material when the deposition rate was calibrated, with just one source started on, and during the deposition process, when both the sources were started on. In particular, the deposition rate can be influenced by various parameters, such as the deposition pressure and plasma composition, and their effects are not the same for all elements and compounds. For example, the presence of copper and chromium atoms in plasma can reduce the sputtering yield, leading to a decrease in the sputtering efficiency and, consequently, altering the final composition of the TFs. The obtained samples, which were expected to exhibit similar amounts of copper and chromium, show comparable compositions without significant differences due to the deposition temperature. In addition, the composition obtained allows us to investigate, as proposed, thin films (TFs) with both high and low copper content.

For the dealloying procedure, the samples were dipped in the nitric acid solution, as was described in the Section 2, for one hour and, after the determination of the chemical composition, for another 23 h, reaching a total of 24 h of immersion. In Table 3, the results of the analysis of the chemical composition obtained with EDX analysis are reported.

Already after one hour, under the applied conditions, the samples obtained at room temperature show evident delamination phenomena, leading to the complete detachment of the thin film from the substrate. The other samples, during the dealloying process, change their color from metallic light gray to dark gray. The composition of the sample Cu30-Cr70 150 remains unchanged both after 1 hour and after 24 h. This behavior can be attributed to the high chromium content that, especially at a lower deposition temperature, restrains the copper diffusion, which is no longer able to form continuous domains. So, after the subitaneous dissolution of the few small copper domains located directly on the surface, the corrosion mechanism stops finding extremely finely divided domains admixed with equally finely divided domains of chromium that is, in addition, passivated and protected once in contact with the acidic oxidizing, preventing any further dissolution of copper. The two samples with the composition Cu50-Cr50, both deposited at 150 °C and 300 °C, after one hour of dealloying show a decrease in copper and a consequent increase in chromium. Finally, only the sample Cu50-Cr50 300 shows an ulterior decrease in the copper amount after 24 h of dealloying. In these two samples, the high amount of copper allows the start of the dissolution of copper in the acid solution, but probably, the higher deposition temperature of the deposit obtained at 300 °C gives the atoms the energy needed for the formation of bigger domains of copper and if compared to the ones formed in the sample deposited at 150 °C. Because of the sample Cu50-Cr50 150, the dissolution of copper starts but, being as the copper domains fragmented, the process is significantly slowed down, not being able to produce a detectable progression over time, also for the substantial stability of chromium that when passivated protects the copper standing below. On the sample Cu50-Cr50 300 instead, also after the dissolution of the copper located closer to the surface, the selective dissolution continues for all 24 h.

### 3.2. XPS Analysis

**Figure 2:**
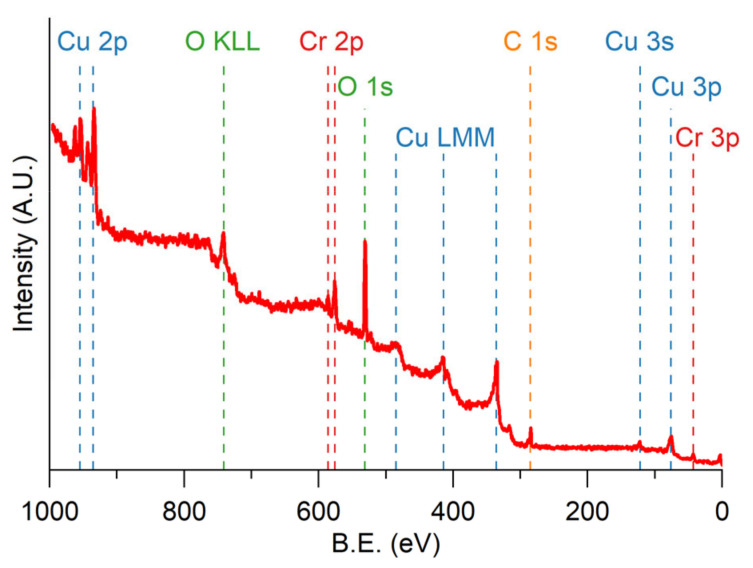
XPS survey spectrum of the sample Cu50-Cr50 150 with the association of each peak to the corresponding transition.

To verify our hypothesis concerning the formation of the passivated chromium oxide layer, XPS analysis was performed. This kind of analysis permits us to obtain information from the first few angstroms (from 10 Å to 15 Å, or from 1 nm to 1.5 nm) due to the low mean free path of the photoemitted electrons in the solid matter. In Figure 2, the survey spectrum of the sample Cu50-Cr50 150 is reported as an example, with the association of each peak to the corresponding transition.

From the analysis of the spectrum, no elements other than those expected are present.

**Figure 3:**
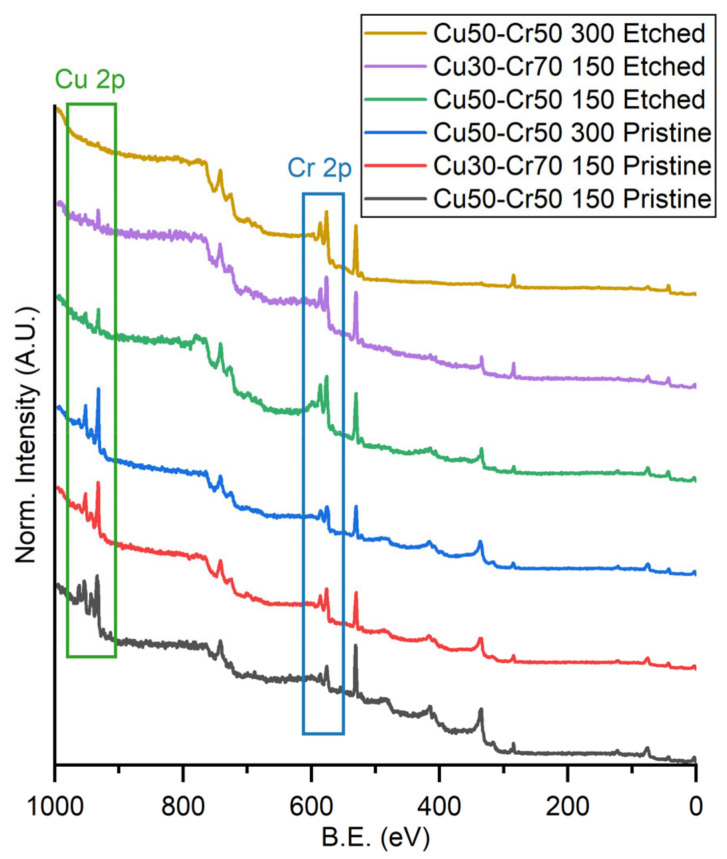
Comparison of the XPS survey spectra of the three samples before and after the dealloying process (etched). The green box highlights the Cu 2p region and the blue box highlights the Cr 2p region.

In Figure 3, the spectra of the three samples are shown for comparison, both before and after the dealloying process, together with two boxes that highlight the Cu 2p and the Cr 2p regions.

From Figure 3, it is possible to understand that the elements present in the different samples are the same. It is also possible to understand the general trend in the copper and chromium amounts in the samples: the copper peaks of the sample Cu50-Cr50 150 are slightly different from the ones of the other two samples before the dealloying process, showing an evident double doublet, due to the presence of shake-up peaks. After dealloying, the intensity of the copper peaks in general decreases while chromium increases, evident confirmation that the dealloying process works properly.

In Table 4, the compositions of the samples before and after the dealloying process determined with XPS analysis are restated and compared with the ones determined with EDX analysis.

The quantification results obtained with XPS and EDX are similar for all the samples before the dealloying process. As confirmation of the hypothesis of the formation of the passive layer of chromium oxide during the dealloying process, the surface of all the samples presents a high concentration of chromium (percentage related to the XPS analysis) compared to the bulk composition, and they show a resulting deficiency in the amount of copper and, as depicted in Figure 3, the samples after etching are characterized by an increased quantity of oxidized chromium.

**Figure 4:**
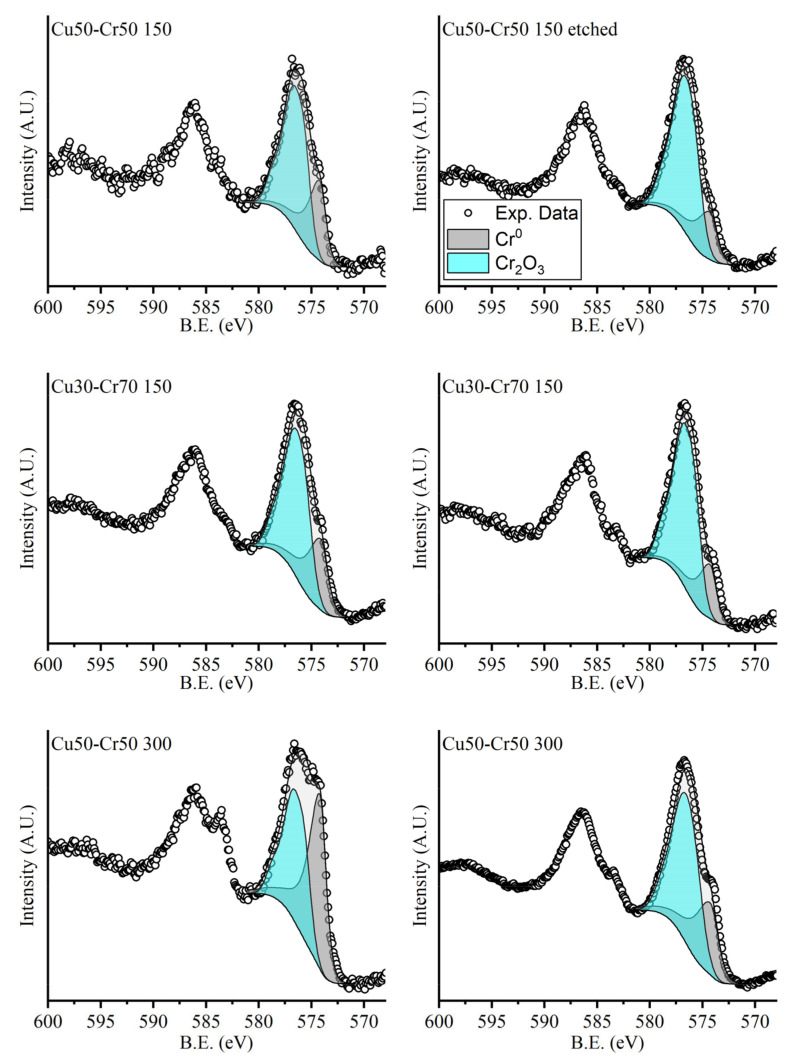
High-resolution spectra acquired in the region of the 2p transition of chromium. The peaks were fitted with two components: the gray one attributed to metallic chromium and the blue one attributed to chromium oxide.

**Figure 5:**
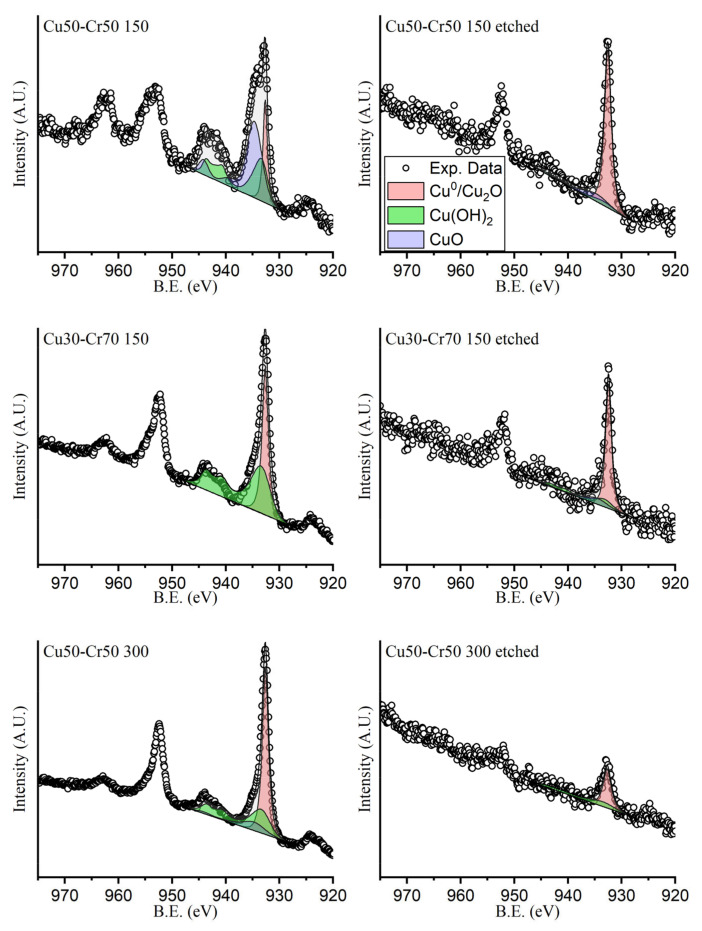
High-resolution spectra acquired in the region of the 2p transition of copper. The peaks were fitted with three components: the red one attributed to metallic copper or copper (I) oxide, the green one attributed to copper hydroxide and the violet one attributed to copper (II) oxide.

As further evidence of the proposed hypothesis, the high-resolution XPS spectra of the 2p transitions of chromium and copper are presented below, respectively, in Figure 4 and Figure 5. The intensities were normalized to allow for the comparison of the spectra of the different samples, not in terms of the relative abundance between samples but in terms of the shape of the peaks.

All the components used for the fitting of the peaks of both chromium and copper are the result of the sum of more than one mixed Gaussian–Lorentzian curve. All the curves used for the same compound were related to each other in terms of the relative positions, areas and full widths at half maximum (FWHMs), following the indication reported in the literature by Biesinger et al. [39,40]. In Figure 4 and Figure 5, the normalized spectra are shown, making it possible to compare just the position and the shape of the peaks, not their intensities.

In Figure 4, it is possible to note that the relative amounts of metallic chromium and chromium oxide do not change significantly in the various spectra due to the native oxide formation on the surface of the sample. The only sample that shows a different composition is the Cu50-Cr50 300 one, which has higher concentrations of chromium oxide, probably due to the high temperature of deposition that brings about the formation of a thick layer of oxide directly in the instrument.

**Figure 6:**
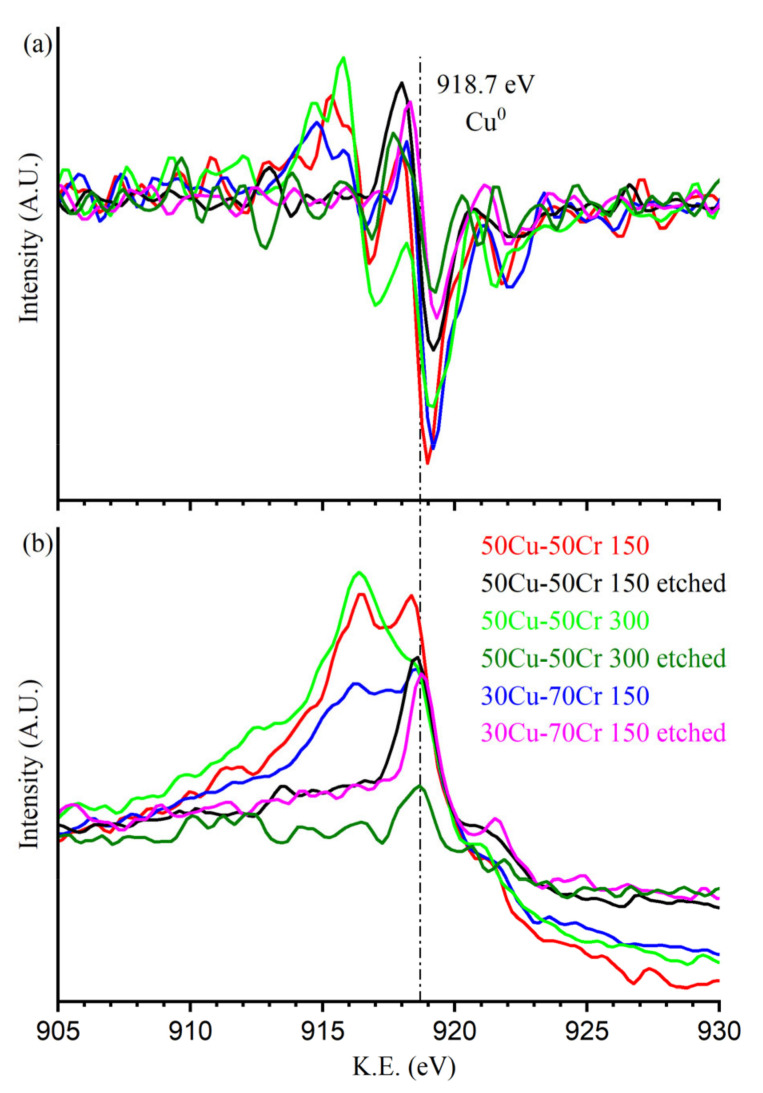
High-resolution spectra in the region of the Auger peak of copper (**a**) before and (**b**) after differentiation. The vertical line marks the position of the peak relative to the metallic copper at 918.7 eV.

In Figure 5, it is possible to note that all the fully oxidized components of copper (CuO and Cu(OH)_2_), likely located on the outer surface of the sample due to copper’s natural tendency to oxidize, are removed during the dealloying process. This fact is evidenced both from the fitting and, as confirmation, from the disappearance of the shake-up peaks (the two broadened peaks at about 943 eV and 963 eV) typically present in the compound of copper (II). On the other hand, the metallic or partially oxidized copper (Cu^0^/Cu_2_O) was removed only in the sample Cu50-Cr50 300, where the dealloying solution can reach the inner part of the deposited film, thanks to the continuity of the larger copper domain that protrudes more deeply. In any case, as it is possible to see by matching the data from the Cu 2p photoelectron line and the Auger peak reported in Figure 6, the stable presence of metallic copper after the dealloying process suggests that copper is located below the passivating layer of chromium oxide and not exposed to the oxygen in the atmosphere, which can easily lead to the complete oxidation of copper. The suggested presence of metallic copper in the as-prepared sample can be proof of a certain degree of admixing of the deposit, which might have led to the formation of an alloy with a solubility of copper in chromium that is sensibly lower, even at a higher temperature, with the canonical methods employed to produce alloys [34].

With XPS peak fitting, it is impossible to distinguish copper (I) oxide from metallic copper, but the speciation can be obtained with the analysis of the Auger peak, which in our case gives us really interesting information. In fact, before the selective dissolution process (red, light green and blue lines), the presence of more than one maximum results in the attribution of the presence of copper one and copper two that, along with the information from the photoelectron line, can be found both the metallic copper and, located at lower kinetic energy values, the change in slope of the Auger peak due to the presence of copper (I). After the selective dissolution process, in samples 50Cu-50Cr 150 and 30Cu-70Cr 150 (black and pink lines), the components at lower kinetic energy disappear, leaving the metallic copper contribution. Finally, for the sample 50Cu-50Cr 300, after the selective dissolution process, both contributions at lower kinetic energy disappear and the one attributed to metallic copper significantly decreases (dark green line).

In Table 5, the relative percentage of each component is reported to better understand the differences between the samples before and after the acid dissolution process.

The percentages reported in Table 5 confirm that, after the selective dissolution process, the chromium shows an increase in the oxide to metal ratio, while the copper hydroxide shows a marked decrease of its Cu(II) form and a dramatic increase of Cu(0).

### 3.3. XRD Analysis

To investigate the lattice structure of the TFs, the XRD diffraction patterns were acquired in Brag–Brentano mode on the samples deposited on silicon wafer, to prevent the interferences caused by the chromium and iron in the stainless steel.

**Figure 7:**
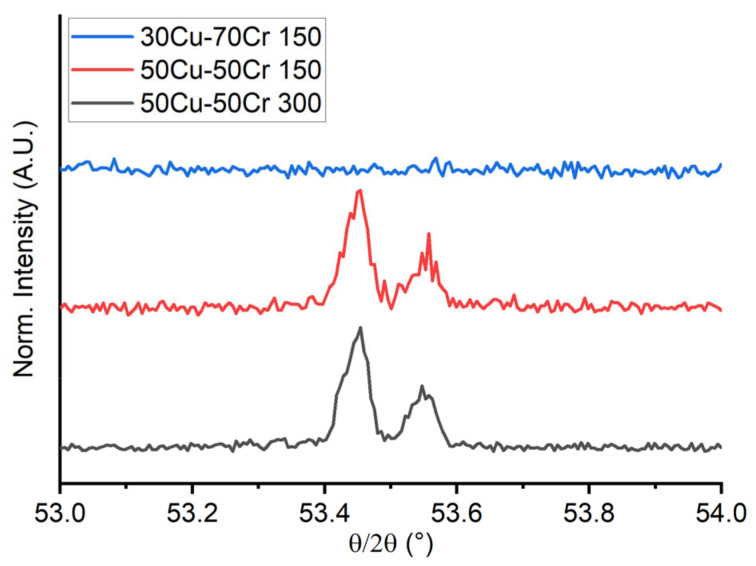
Comparison of the high-resolution XRD diffraction patterns of the region of the {200} reflex of copper for the three samples.

In the samples with a composition of 50 wt.% copper and 50 wt.% chromium, both deposited at 150 °C and 300 °C, the {200} diffraction peak of copper was recognized, but we did not detect any peak attributable to chromium, meaning that chromium was deposited in a quasi-amorphous structure. This is a finding that can be reasonably explained with the higher melting temperature of chromium and the higher tendency to form solid deposits without long-range ordering when realized at low temperature. In the sample 30Cu-70Cr 150, no diffraction peaks attributable to the thin film were recognized, probably due to the low content of copper that might be an obstacle by the high quantity of chromium in the coarsening process and not being able to form bigger domains. In Figure 7 is reported the comparison of the high-resolution XRD diffraction patterns of the region of the {200} reflex of copper for the three samples.

**Figure 8:**
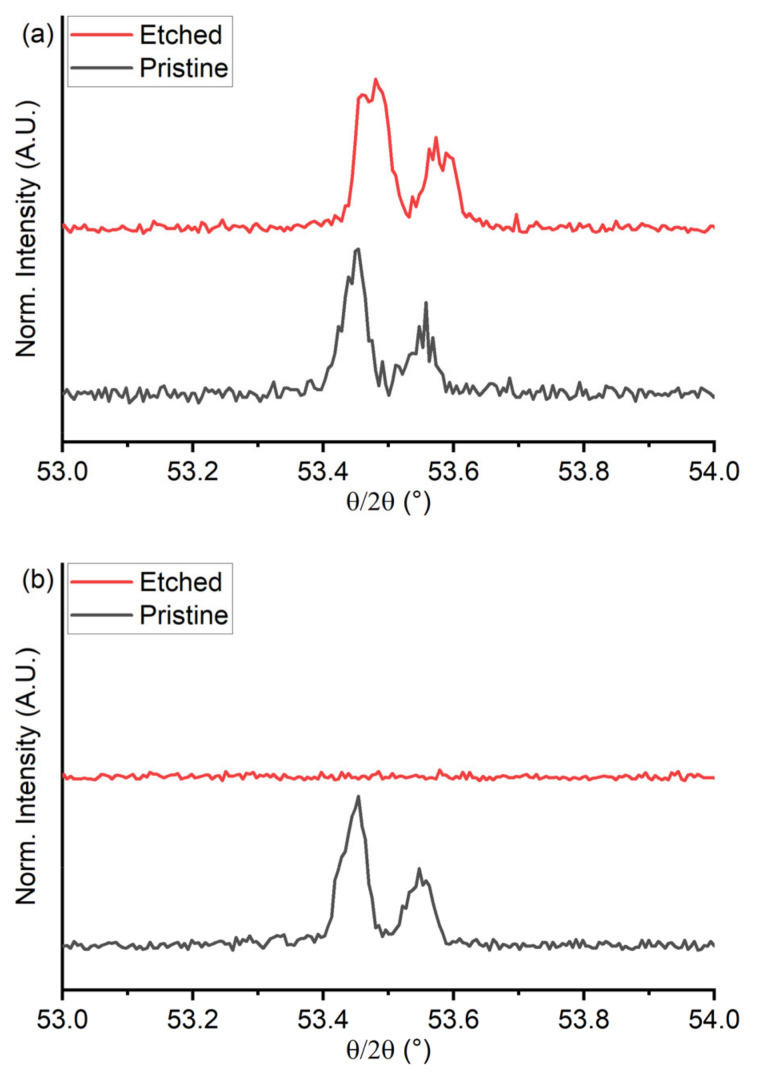
Comparison of the high-resolution XRD diffraction patterns of the region of the {200} reflex of copper before and after the dealloying process for the samples (**a**) 50Cu-50Cr 150 and (**b**) 50Cu-50Cr 300.

In Figure 8 are reported the comparisons of the high-resolution XRD diffraction patterns of the {200} reflex of the copper region before and after the selective dissolution. In Figure 8b, it is possible to note that the reflex disappears after the dealloying process in sample 50Cu-50Cr 300, confirming the removal of the copper from the thin film; quite the opposite, the reflex remains in the sample 50Cu-50Cr 150 (Figure 8a).

### 3.4. SEM Analysis

**Figure 9:**
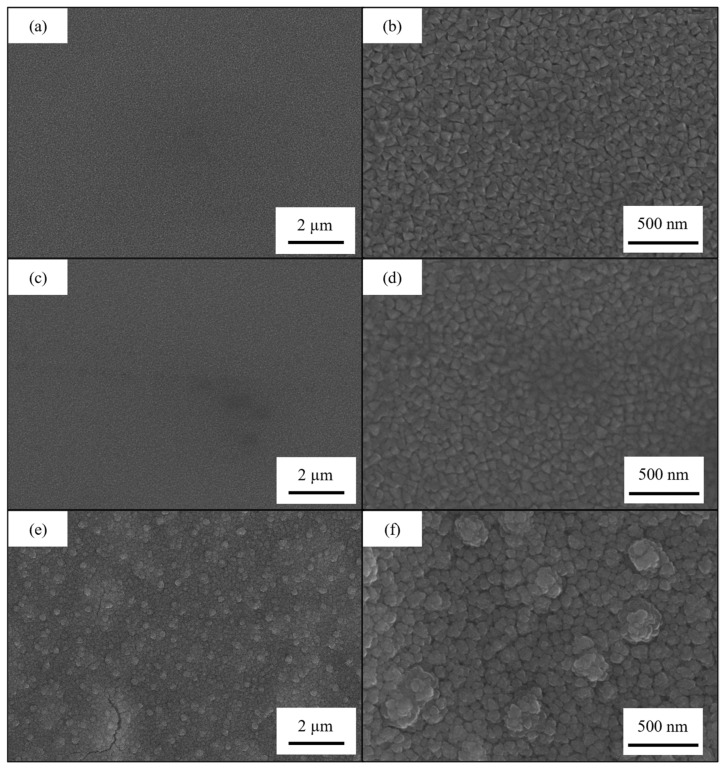
SEM scans of the samples (**a**,**b**) 30Cu-70Cr 150, (**c**,**d**) 50Cu-50Cr 150 and (**e**,**f**) 50Cu-50Cr 300 acquired at a magnification of (**a**,**c**,**e**) ×10k and (**b**,**d**,**f**) ×50k.

To investigate the surface morphology of the samples, SEM images were acquired. In Figure 9 are reported the scans for all the samples at a magnification of ×10k and ×50k before the dealloying process.

**Figure 10:**
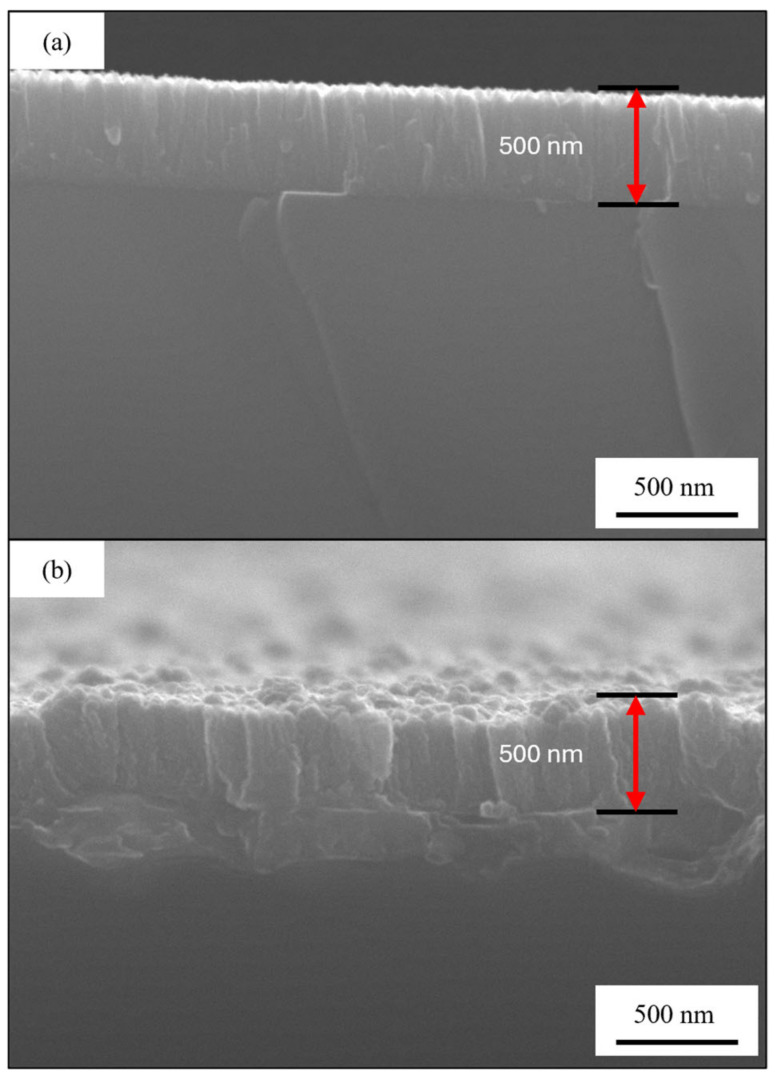
Comparison of the cross-section images of the samples (**a**) 30Cu-70Cr 150 and (**b**) 50Cu-50Cr 300 at a magnification of ×40k.

The samples 30Cu-70Cr 150 (Figure 9a,b) and 50Cu-50Cr 150 (Figure 9c,d) show similar surface morphology, both in terms of the dimension (about 50 nm of diameter) and the shape of the crystals (polygonal). The sample 50Cu-50Cr 300 (Figure 9e,f) shows a different structure, with bigger crystals (about 100 nm of diameter), and a different shape (more spherical). This difference can probably be attributed to the different temperatures during growth and the different degrees of oxidation. To better evaluate the difference between the samples obtained at different temperatures, the silicon wafer was broken following the crystal plane of the substrate, obtaining a brittle fracture that let us acquire an image of the cross-section. In Figure 10, the cross-section images of samples 30Cu-70Cr 150 (Figure 10a) and 50Cu-50Cr 300 (Figure 10b) are reported.

**Figure 11:**
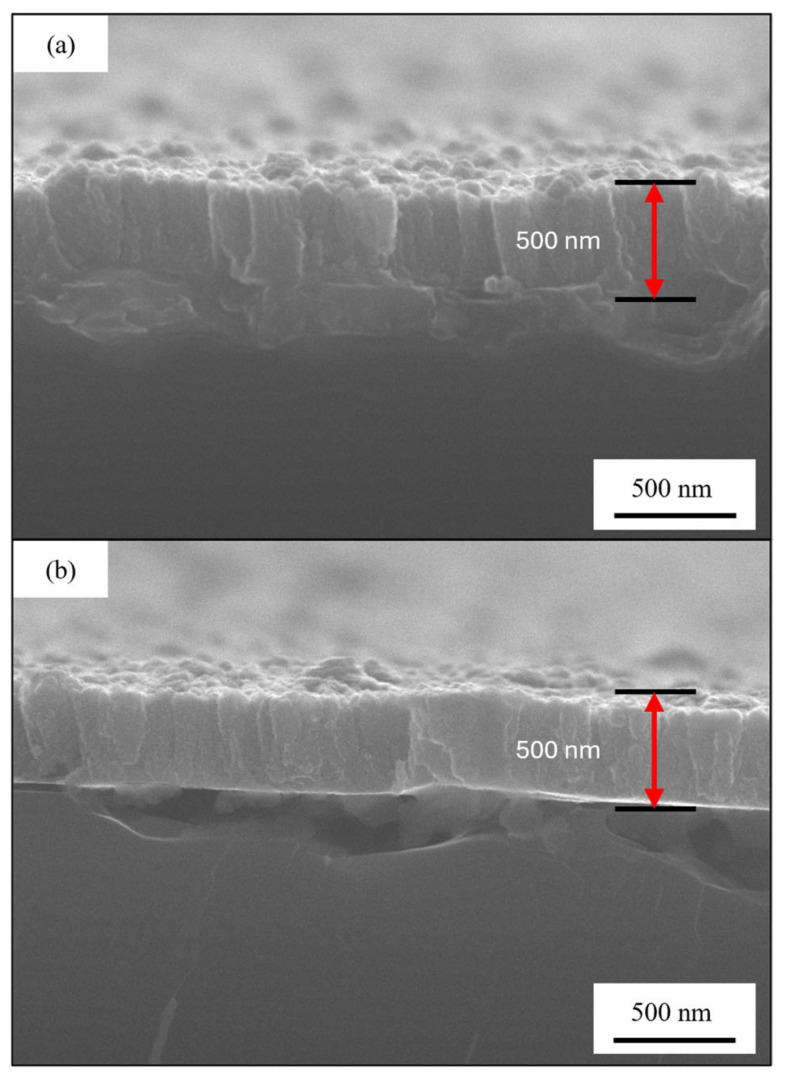
Comparison of the cross-section images for the sample 50Cu-50Cr 300 (**a**) before and (**b**) after the selective dissolution process at a magnification of ×40k.

The cross-section images confirm that the dimensions of the crystals in the sample grown at 300 °C are bigger than the one grown at 150 °C. Analyzing this images, it is possible to observe the typical columnar grow of the PVD samples, and it is possible also to confirm that the thickness of the films is the expected one, for the samples grown at both 150 °C and 300 °C. Finally, in Figure 11 are presented the images of the cross-section before (Figure 11a) and after (Figure 11b) the dealloying process for the sample 50Cu-50Cr 300.

The cross-section after the dealloying process does show a change in the appearance of the coating, since the marked presence of the columnar growth seems to disappear after the etching step, while the total thickness is unchanged, giving us the idea of a process that starting from the surface has proceeded inward of the coating, leading to the formation of voids. The results suggest that the low amount of copper that is left and found to be metallic during the XPS analysis could be due to the portion of copper that effectively formed an alloy with chromium. The other part, the one that has been easily removed by the acid treatment, is probably the one that is not participating in the alloy formation.

### 3.5. TEM Analysis

**Figure 12:**
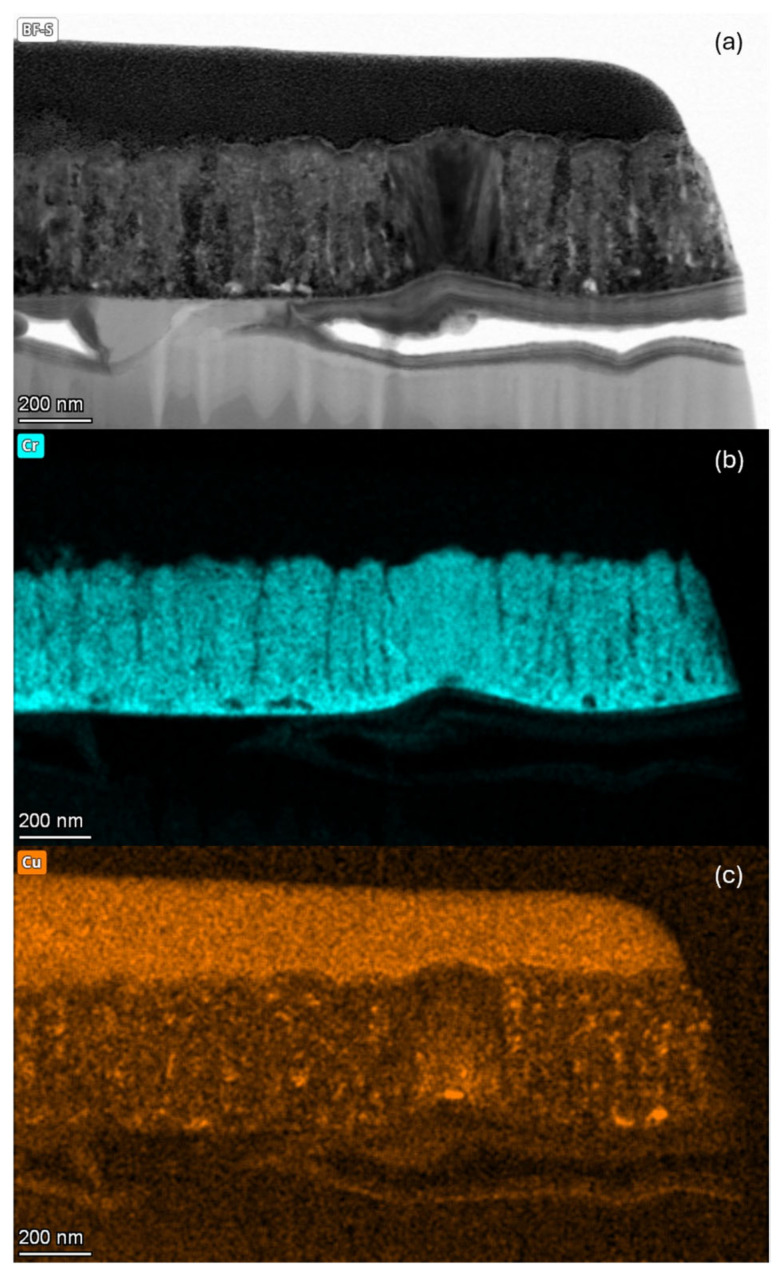
(**a**) TEM analysis of the cross-section of sample 50Cu-50Cr 300 before the selective dissolution process and distribution of (**b**) chromium and (**c**) copper.

Figure 12 shows the TEM analysis of the cross-section of the 50Cu-50Cr 300 sample. To obtain the image, lamella preparation was performed using a Tescan GAIA 3 FIB/SEM microscope, equipped with a Ga ion column. A 30 keV Gallium ion beam was used for the milling procedure, while the final polishing/de-amorphization was carried out at 5 keV. The final lamella thickness was below 50 nm. A protective layer was first deposited from an organometallic Pt precursor on the surface of the sample to avoid dulling of the film cross-section during ion beam etching. This platinum layer, which is not reported in the following STEM EDX maps, was produced in two steps: (a) the first thin Pt deposit achieved by electron beam at 5 kV and (b) a second massive 2 µm Pt deposit.

From the analysis of Figure 12a, the structure of the obtained TFs after the selective dissolution of copper can be observed. The film exhibits fine porosity, evenly distributed throughout the entire thickness of the films, along with larger vertical channels, which seem to cross almost all the thickness of the layer. Another interesting observation concerns the residual copper (Figure 12c), which appears enclosed in small and evenly distributed domains inside the film section. We could attribute the stronger Cu signal on the protective Pt layer to the experimental apparatus. The lamella holder is in fact made of copper. The stronger Cu signal in concurrence with the protective layer could be associated with the interaction of the electrons scattered from a heavier Pt with the sample’s near environment (lamella holder). Finally, the chromium distribution (Figure 12c) is uniform across the entire thickness of the thin films.

**Figure 13:**
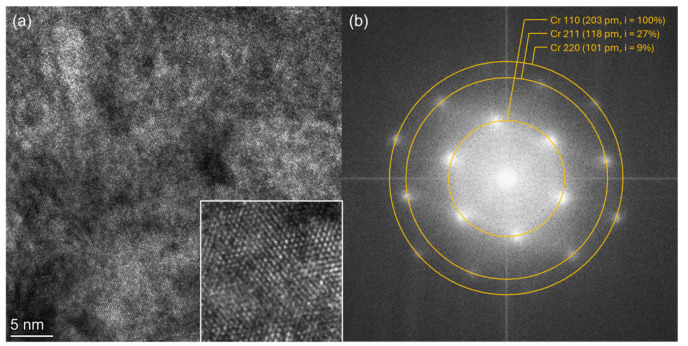
(**a**) HR-TEM image showing the presence of a fine structure in the form of lattice fringes and (**b**) FFT analysis of the image.

The HR-TEM images acquired of the TF portion of the lamella evidenced the presence of a crystallographic order in the form of lattice fringes (Figure 13a). By converting the image into the frequency domain using the fast Fourier transform (FFT), different signals appear. An analysis of the FFT highlights the presence of cubic Cr(0). By comparing the simulated diffractograms from the Inorganic Crystalline Structure Database (ICSD) for cubic Cr (1751627) and cubic Cu (1765816), it is in fact possible to define the presence of Cr 110 (203 pm, i = 100%), Cr 211 (118 pm, i = 27%) and Cr 220 (101 pm, i = 9%) (Figure 13b). The Cu 111 (208 pm, i = 100%) and Cu 222 (104 pm, i = 8%) that fell near could be excluded, together with the Cu 220 (128 pm, i = 24%). Still, we were not able to acquire an image in an area in which we were certain of the presence of the Cu phase.

### 3.6. Surface Characterization

As the final characterization of the samples’ surface, the wettability analysis was performed on TFs grown on stainless steel before and after the dealloying process. The results of the contact angle measurements are reported in Table 6.

From the data reported in Table 6, it is possible to note that before the dealloying process, the contact angle of the water drops decreases before the application of the TFs of the copper–chromium alloy, probably due to the presence of copper oxide on the surface. After the dealloying process, the wettability increases dramatically, proving the formation of a chromium oxide film with different properties compared to the native chromium oxide layer naturally present on the surface of stainless steel. This is an attended result since we performed a wet acid dissolution, being able to solubilize only the copper that can be reached by the oxidizing agent and hydrogen ions through the medium (water) [41].

## 4. Conclusions

This research successfully demonstrated the potential of Cu–Cr alloy dealloying for fabricating nanostructured chromium TFs. This study emphasized the critical influence of the deposition temperature and film composition on the morphology and chemical properties. Higher deposition temperatures resulted in improved porosity, promoting the formation of larger, well-separated copper and chromium domains. After dealloying, these larger domains led to increased surface porosity, which enhanced the wettability and facilitated the formation of more hydrophilic compounds on the surface.

The relative content of copper and chromium played a more complex role, influencing the success or failure of atomic coarsening, as suggested by the XRD diffractogram. A film composed of 66 wt.% copper and 34 wt.% chromium, deposited at 300 °C, proved optimal for obtaining a nanostructured chromium TF. After 24 h in a nitric acid solution, up to 95% of the copper was removed, leaving behind fine, evenly distributed porosity throughout the film thickness, along with larger vertical channels traversing almost the entire layer, as observed in the TEM analysis.

These findings highlight the potential of such films for advanced applications, such as catalysis, energy storage, and biomedicine. The ability to tailor the surface properties by simply removing copper from a Cu–Cr thin film produced via magnetron sputtering is particularly promising. As is well known, the surface processes and the effectiveness of these applications are enhanced by increased surface area. Furthermore, improved wettability promotes greater interaction between the material surface and aqueous solutions, further boosting the activity of the nanostructured material.

Future work will aim to optimize the dealloying process and explore additional functional applications to further expand the utility of these films.

## Figures and Tables

**Table 1:** Sample characteristics.

Num.	Cu–Cr (wt.%)	Substrate Temp.	RF Power (Cr)	DC Current (Cu)
1	50–50%	R.T.	150 W	130 mA
2	30–70%	R.T.	150 W	80 mA
3	50–50%	150 °C	150 W	130 mA
4	30–70%	150 °C	150 W	80 mA
5	50–50%	300 °C	150 W	130 mA
6	30–70%	300 °C	150 W	80 mA

**Table 2:** TF compositions determined with EDX analysis compared to the expected ones.

Sample	EDX (wt.%)		Expected (wt.%)	
	Cu	Cr	Cu	Cr
Cu50-Cr50 RT	75%	25%	50%	50%
Cu50-Cr50 150	67%	33%	50%	50%
Cu50-Cr50 300	66%	34%	50%	50%
Cu30-Cr70 RT	43%	57%	30%	70%
Cu30-Cr70 150	53%	47%	30%	70%
Cu30-Cr70 300	N.A.	N.A.	30%	70%

**Table 3:** TF composition determined with EDX analysis before the dealloying procedure, after 1 h and after 24 h.

Sample	Element	As Prepared (wt.%)	1 h (wt.%)	24 h (wt.%)
Cu50-Cr50 RT	Cu	75%	N.A.	N.A.
	Cr	25%	N.A.	N.A.
Cu50-Cr50 150	Cu	67%	52%	52%
	Cr	33%	48%	48%
Cu50-Cr50 300	Cu	66%	49%	17%
	Cr	34%	51%	83%
Cu30-Cr70 RT	Cu	43%	N.A.	N.A.
	Cr	57%	N.A.	N.A.
Cu30-Cr70 150	Cu	53%	48%	48%
	Cr	47%	52%	52%
Cu30-Cr70 300	Cu	N.A.	N.A.	N.A.
	Cr	N.A.	N.A.	N.A.

**Table 4:** TF compositions determined with XPS and compared with the ones determined with EDX analysis before the dealloying procedure and after 24 h.

Sample	Element	As Prepared		24 h	
		XPS (wt.%)	EDX (wt.%)	XPS (wt.%)	EDX (wt.%)
Cu50-Cr50 150	Cu	73%	67%	19%	52%
	Cr	27%	33%	81%	48%
Cu50-Cr50 300	Cu	60%	66%	5%	17%
	Cr	40%	34%	95%	83%
Cu30-Cr70 150	Cu	54%	53%	16%	48%
	Cr	46%	47%	84%	52%

**Table 5:** TF compositions determined by means of XPS and compared with the ones determined with EDX analysis before (“B.” columns) and after (“A.” columns) the dealloying procedure of 24 h.

Sample	Cu						Cr			
	Cu^0^		Cu(OH)_2_		CuO		Cr^0^		Cr_2_O_3_	
	B. (wt.%)	A. (wt.%)	B. (wt.%)	A. (wt.%)	B. (wt.%)	A. (wt.%)	B. (wt.%)	A. (wt.%)	B. (wt.%)	A. (wt.%)
Cu50-Cr50 150	15%	74%	56%	18%	29%	8%	31%	23%	69%	77%
Cu50-Cr50 300	54%	66%	13%	0%	33%	34%	53%	33%	47%	67%
Cu30-Cr70 150	42%	66%	1%	16%	57%	18%	31%	23%	69%	77%

**Table 6:** Results of the contact angle measurements performed on the samples before and after the selective dissolution process.

Sample	Dealloying Process	
	Before	After
Cu30-Cr70 150	50°	0°
Cu50-Cr50 150	44°	0°
Cu50-Cr50 300	55°	0°
Stainless steel	65°	N.A.

## Data Availability

The original contributions presented in this study are included in the article. Further inquiries can be directed to the corresponding author.
